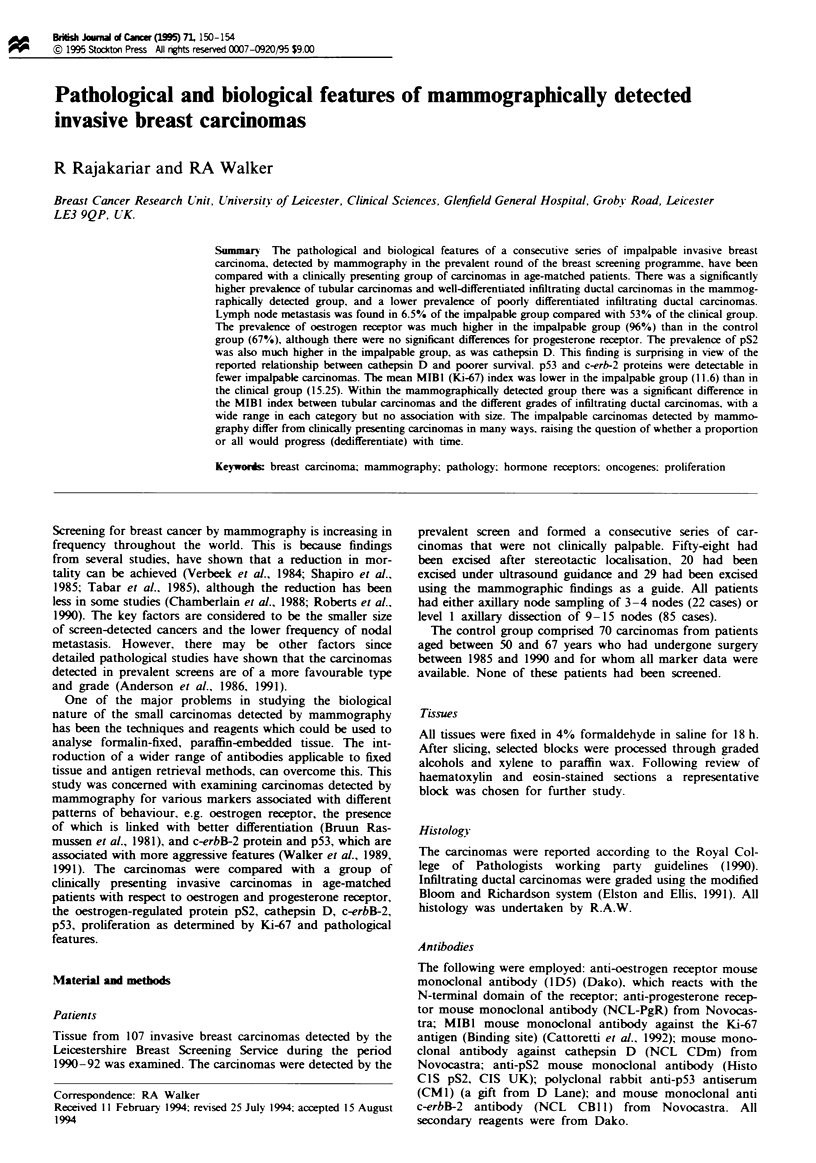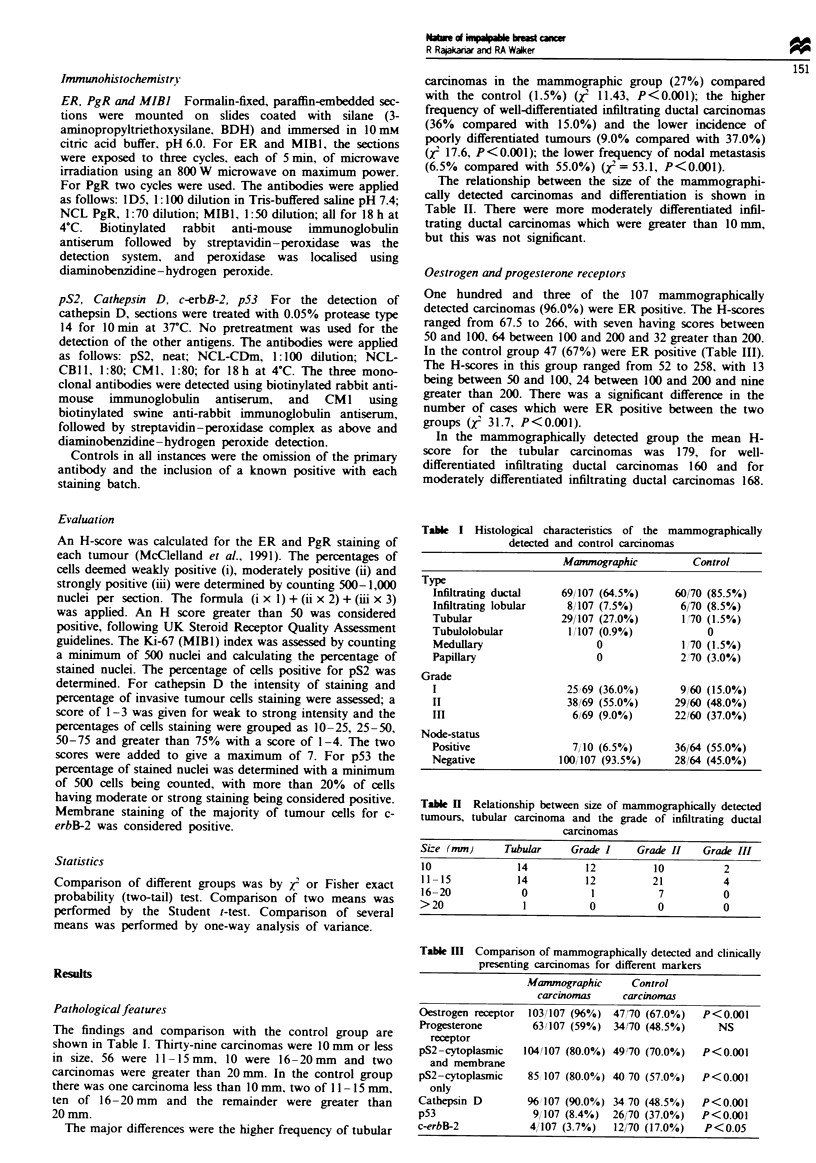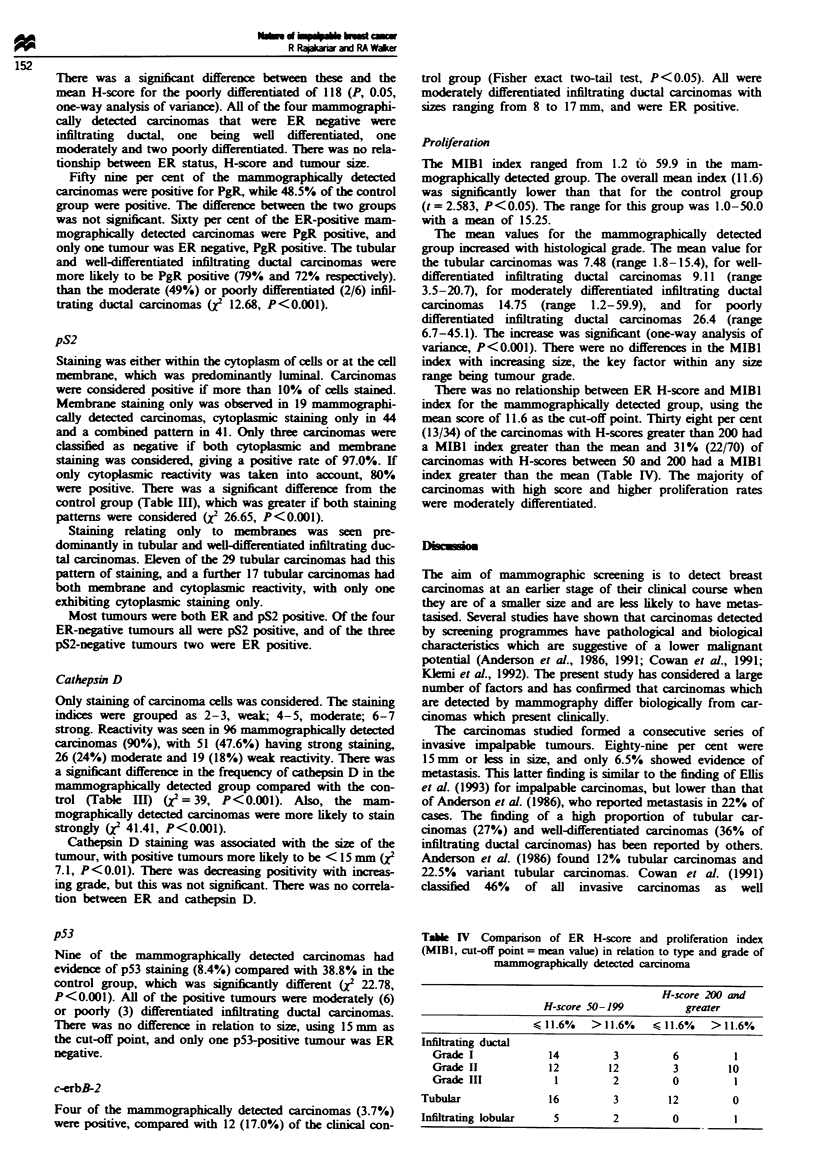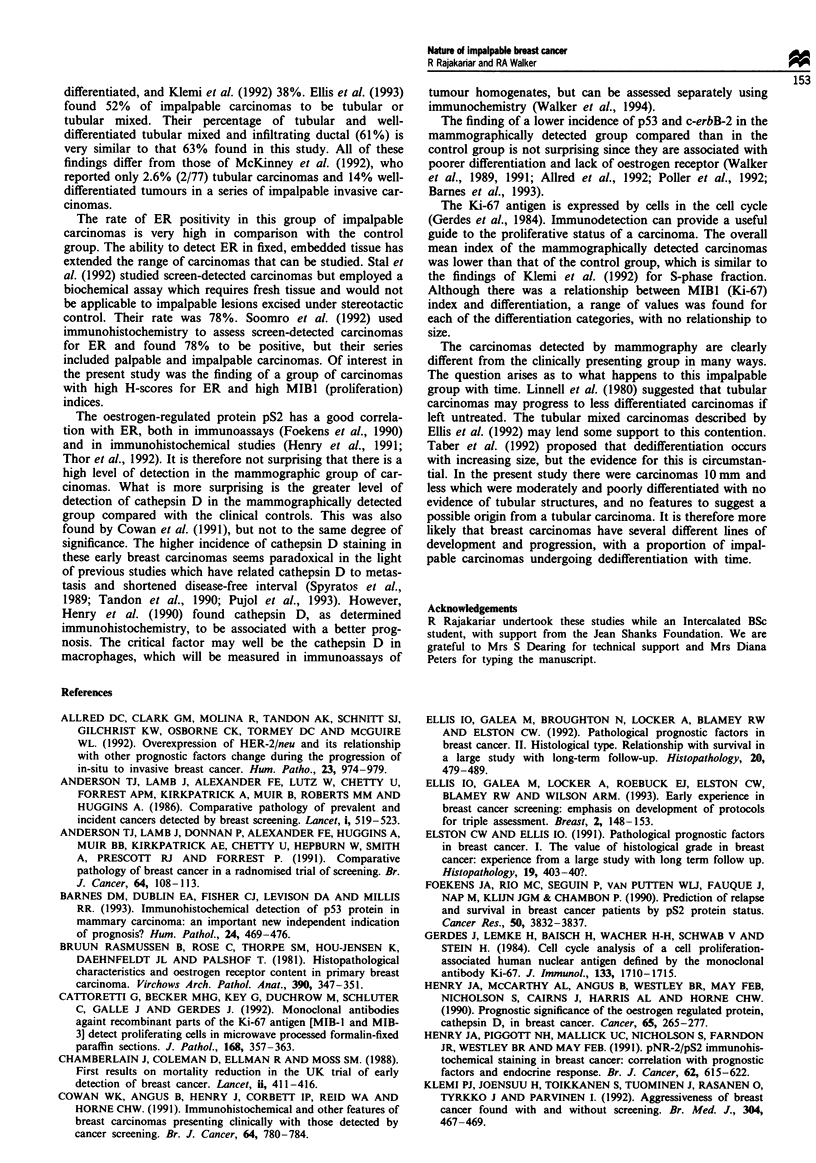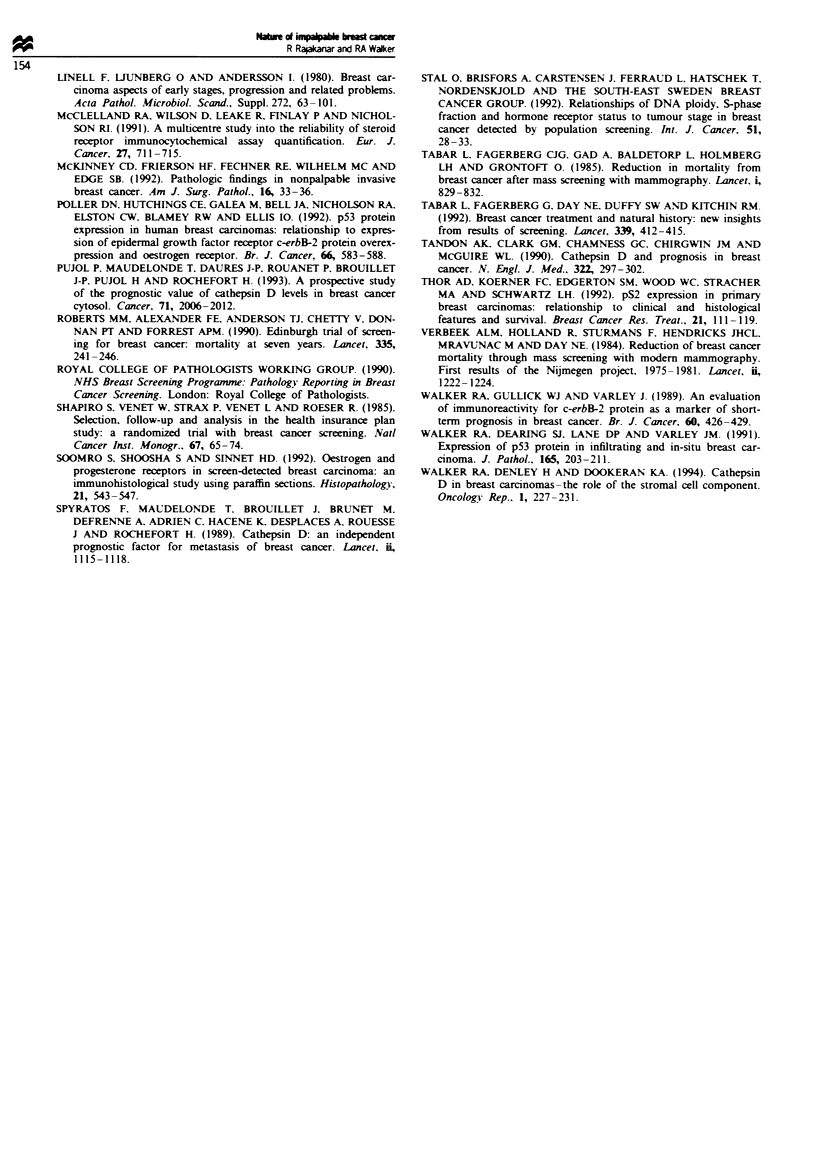# Pathological and biological features of mammographically detected invasive breast carcinomas.

**DOI:** 10.1038/bjc.1995.31

**Published:** 1995-01

**Authors:** R. Rajakariar, R. A. Walker

**Affiliations:** Breast Cancer Research Unit, University of Leicester, UK.

## Abstract

The pathological and biological features of a consecutive series of impalpable invasive breast carcinoma, detected by mammography in the prevalent round of the breast screening programme, have been compared with a clinically presenting group of carcinomas in age-matched patients. There was a significantly higher prevalence of tubular carcinomas as well-differentiated infiltrating ductal carcinomas in the mammographically detected group, and a lower prevalence of poorly differentiated infiltrating ductal carcinomas. Lymph node metastasis was found in 6.5% of the impalpable group compared with 53% of the clinical group. The prevalence of oestrogen receptor was much higher in the impalpable group (96%) than in the control group (67%), although there were no significant differences for progesterone receptor. The prevalence of pS2 was also much higher in the impalpable group, as was cathepsin D. This finding is surprising in view of the reported relationship between cathepsin D and poorer survival. p53 and c-erb-2 proteins were detectable in fewer impalpable carcinomas. The mean MIBI (Ki-67) index was lower in the impalpable group (11.6) than in the clinical group (15.25). Within the mammographically detected group there was a significant difference in the MIBI index between tubular carcinomas and the different grades of infiltrating ductal carcinomas, with a wide range in each category but no association with size. The impalpable carcinomas detected by mammography differ from clinically presenting carcinomas in many ways, raising the question of whether a proportion or all would progress (dedifferentiate) with time.


British Journal of Cancer (1995) 71, 150-154

© 1995 Stockton Press All rights reserved 0007-0920/95 $9.00

### Pathological and biological features of mammographically detected invasive breast carcinomas

R Rajakariar and RA Walker

*Breast Cancer Research Unit, University of Leicester, Clinical Sciences, Glenfield General Hospital, Groby Road, Leicester LE3 9QP, UK.*

**Summary** The pathological and biological features of a consecutive series of impalpable invasive breast carcinoma, detected by mammography in the prevalent round of the breast screening programme, have been compared with a clinically presenting group of carcinomas in age-matched patients. There was a significantly higher prevalence of tubular carcinomas and well-differentiated infiltrating ductal carcinomas in the mammographically detected group, and a lower prevalence of poorly differentiated infiltrating ductal carcinomas. Lymph node metastasis was found in 6.5% of the impalpable group compared with 53% of the clinical group. The prevalence of oestrogen receptor was much higher in the impalpable group (96%) than in the control group (67%), although there were no significant differences for progesterone receptor. The prevalence of pS2 was also much higher in the impalpable group, as was cathepsin D. This finding is surprising in view of the reported relationship between cathepsin D and poorer survival. p53 and c-*erb*-2 proteins were detectable in fewer impalpable carcinomas. The mean MIB1 (Ki-67) index was lower in the impalpable group (11.6) than in the clinical group (15.25). Within the mammographically detected group there was a significant difference in the MIB1 index between tubular carcinomas and the different grades of infiltrating ductal carcinomas, with a wide range in each category but no association with size. The impalpable carcinomas detected by mammography differ from clinically presenting carcinomas in many ways, raising the question of whether a proportion or all would progress (dedifferentiate) with time.

**Keywords:** breast carcinoma; mammography; pathology; hormone receptors; oncogenes; proliferation

Screening for breast cancer by mammography is increasing in frequency throughout the world. This is because findings from several studies, have shown that a reduction in mortality can be achieved (Verbeek *et al.*, 1984; Shapiro *et al.*, 1985; Tabar *et al.*, 1985), although the reduction has been less in some studies (Chamberlain *et al.*, 1988; Roberts *et al.*, 1990). The key factors are considered to be the smaller size of screen-detected cancers and the lower frequency of nodal metastasis. However, there may be other factors since detailed pathological studies have shown that the carcinomas detected in prevalent screens are of a more favourable type and grade (Anderson *et al.*, 1986, 1991).

One of the major problems in studying the biological nature of the small carcinomas detected by mammography has been the techniques and reagents which could be used to analyse formalin-fixed, paraffin-embedded tissue. The introduction of a wider range of antibodies applicable to fixed tissue and antigen retrieval methods, can overcome this. This study was concerned with examining carcinomas detected by mammography for various markers associated with different patterns of behaviour, e.g. oestrogen receptor, the presence of which is linked with better differentiation (Bruun Rasmussen *et al.*, 1981), and c-*erb*B-2 protein and p53, which are associated with more aggressive features (Walker *et al.*, 1989, 1991). The carcinomas were compared with a group of clinically presenting invasive carcinomas in age-matched patients with respect to oestrogen and progesterone receptor, the oestrogen-regulated protein pS2, cathepsin D, c-*erb*B-2, p53, proliferation as determined by Ki-67 and pathological features.

### Material and methods

### Patients

Tissue from 107 invasive breast carcinomas detected by the Leicestershire Breast Screening Service during the period 1990-92 was examined. The carcinomas were detected by the prevalent screen and formed a consecutive series of carcinomas that were not clinically palpable. Fifty-eight had been excised after stereotactic localisation, 20 had been excised under ultrasound guidance and 29 had been excised using the mammographic findings as a guide. All patients had either axillary node sampling of 3-4 nodes (22 cases) or level 1 axillary dissection of 9-15 nodes (85 cases).

The control group comprised 70 carcinomas from patients aged between 50 and 67 years who had undergone surgery between 1985 and 1990 and for whom all marker data were available. None of these patients had been screened.

### Tissues

All tissues were fixed in 4% formaldehyde in saline for 18 h. After slicing, selected blocks were processed through graded alcohols and xylene to paraffin wax. Following review of haematoxylin and eosin-stained sections a representative block was chosen for further study.

### Histology

The carcinomas were reported according to the Royal College of Pathologists working party guidelines (1990). Infiltrating ductal carcinomas were graded using the modified Bloom and Richardson system (Elston and Ellis, 1991). All histology was undertaken by R.A.W.

### Antibodies

The following were employed: anti-oestrogen receptor mouse monoclonal antibody (1D5) (Dako), which reacts with the N-terminal domain of the receptor; anti-progesterone receptor mouse monoclonal antibody (NCL-PgR) from Novocastra; MIB1 mouse monoclonal antibody against the Ki-67 antigen (Binding site) (Cattoretti *et al.*, 1992); mouse monoclonal antibody against cathepsin D (NCL CDm) from Novocastra; anti-pS2 mouse monoclonal antibody (Histo C1S pS2, CIS UK); polyclonal rabbit anti-p53 antiserum (CM1) (a gift from D Lane); and mouse monoclonal anti c-*erb*B-2 antibody (NCL CB11) from Novocastra. All secondary reagents were from Dako.

Correspondence: RA Walker
Received 11 February 1994; revised 25 July 1994; accepted 15 August 1994

*Immunohistochemistry*

*ER, PgR and MIB1* Formalin-fixed, paraffin-embedded sections were mounted on slides coated with silane (3-aminopropyltriethoxysilane, BDH) and immersed in 10 mM citric acid buffer, pH 6.0. For ER and MIB1, the sections were exposed to three cycles, each of 5 min, of microwave irradiation using an 800 W microwave on maximum power. For PgR two cycles were used. The antibodies were applied as follows: 1D5, 1:100 dilution in Tris-buffered saline pH 7.4; NCL PgR, 1:70 dilution; MIB1, 1:50 dilution; all for 18 h at 4°C. Biotinylated rabbit anti-mouse immunoglobulin antiserum followed by streptavidin–peroxidase was the detection system, and peroxidase was localised using diaminobenzidine–hydrogen peroxide.

*pS2, Cathepsin D, c-erbB-2, p53* For the detection of cathepsin D, sections were treated with 0.05% protease type 14 for 10 min at 37°C. No pretreatment was used for the detection of the other antigens. The antibodies were applied as follows: pS2, neat; NCL-CDm, 1:100 dilution; NCL-CB11, 1:80; CM1, 1:80; for 18 h at 4°C. The three monoclonal antibodies were detected using biotinylated rabbit anti-mouse immunoglobulin antiserum, and CM1 using biotinylated swine anti-rabbit immunoglobulin antiserum, followed by streptavidin–peroxidase complex as above and diaminobenzidine–hydrogen peroxide detection.

Controls in all instances were the omission of the primary antibody and the inclusion of a known positive with each staining batch.

*Evaluation*

An H-score was calculated for the ER and PgR staining of each tumour (McClelland *et al.*, 1991). The percentages of cells deemed weakly positive (i), moderately positive (ii) and strongly positive (iii) were determined by counting 500–1,000 nuclei per section. The formula (i × 1) + (ii × 2) + (iii × 3) was applied. An H score greater than 50 was considered positive, following UK Steroid Receptor Quality Assessment guidelines. The Ki-67 (MIB1) index was assessed by counting a minimum of 500 nuclei and calculating the percentage of stained nuclei. The percentage of cells positive for pS2 was determined. For cathepsin D the intensity of staining and percentage of invasive tumour cells staining were assessed; a score of 1–3 was given for weak to strong intensity and the percentages of cells staining were grouped as 10–25, 25–50, 50–75 and greater than 75% with a score of 1–4. The two scores were added to give a maximum of 7. For p53 the percentage of stained nuclei was determined with a minimum of 500 cells being counted, with more than 20% of cells having moderate or strong staining being considered positive. Membrane staining of the majority of tumour cells for c-*erb*B-2 was considered positive.

*Statistics*

Comparison of different groups was by $\chi^2$ or Fisher exact probability (two-tail) test. Comparison of two means was performed by the Student *t*-test. Comparison of several means was performed by one-way analysis of variance.

### Results

*Pathological features*

The findings and comparison with the control group are shown in Table I. Thirty-nine carcinomas were 10 mm or less in size, 56 were 11–15 mm, 10 were 16–20 mm and two carcinomas were greater than 20 mm. In the control group there was one carcinoma less than 10 mm, two of 11–15 mm, ten of 16–20 mm and the remainder were greater than 20 mm.

The major differences were the higher frequency of tubular carcinomas in the mammographic group (27%) compared with the control (1.5%) ($\chi^2$ 11.43, $P<0.001$); the higher frequency of well-differentiated infiltrating ductal carcinomas (36% compared with 15.0%) and the lower incidence of poorly differentiated tumours (9.0% compared with 37.0%) ($\chi^2$ 17.6, $P<0.001$); the lower frequency of nodal metastasis (6.5% compared with 55.0%) ($\chi^2 = 53.1$, $P<0.001$).

The relationship between the size of the mammographically detected carcinomas and differentiation is shown in Table II. There were more moderately differentiated infiltrating ductal carcinomas which were greater than 10 mm, but this was not significant.

*Oestrogen and progesterone receptors*

One hundred and three of the 107 mammographically detected carcinomas (96.0%) were ER positive. The H-scores ranged from 67.5 to 266, with seven having scores between 50 and 100, 64 between 100 and 200 and 32 greater than 200. In the control group 47 (67%) were ER positive (Table III). The H-scores in this group ranged from 52 to 258, with 13 being between 50 and 100, 24 between 100 and 200 and nine greater than 200. There was a significant difference in the number of cases which were ER positive between the two groups ($\chi^2$ 31.7, $P<0.001$).

In the mammographically detected group the mean H-score for the tubular carcinomas was 179, for well-differentiated infiltrating ductal carcinomas 160 and for moderately differentiated infiltrating ductal carcinomas 168.

**Table I** Histological characteristics of the mammographically detected and control carcinomas

| | *Mammographic* | *Control* |
|---|---|---|
| Type | | |
| Infiltrating ductal | 69/107 (64.5%) | 60/70 (85.5%) |
| Infiltrating lobular | 8/107 (7.5%) | 6/70 (8.5%) |
| Tubular | 29/107 (27.0%) | 1/70 (1.5%) |
| Tubulolobular | 1/107 (0.9%) | 0 |
| Medullary | 0 | 1/70 (1.5%) |
| Papillary | 0 | 2/70 (3.0%) |
| Grade | | |
| I | 25/69 (36.0%) | 9/60 (15.0%) |
| II | 38/69 (55.0%) | 29/60 (48.0%) |
| III | 6/69 (9.0%) | 22/60 (37.0%) |
| Node-status | | |
| Positive | 7/10 (6.5%) | 36/64 (55.0%) |
| Negative | 100/107 (93.5%) | 28/64 (45.0%) |

**Table II** Relationship between size of mammographically detected tumours, tubular carcinoma and the grade of infiltrating ductal carcinomas

| *Size (mm)* | *Tubular* | *Grade I* | *Grade II* | *Grade III* |
|---|---|---|---|---|
| 10 | 14 | 12 | 10 | 2 |
| 11–15 | 14 | 12 | 21 | 4 |
| 16–20 | 0 | 1 | 7 | 0 |
| >20 | 1 | 0 | 0 | 0 |

**Table III** Comparison of mammographically detected and clinically presenting carcinomas for different markers

| | *Mammographic carcinomas* | *Control carcinomas* | |
|---|---|---|---|
| Oestrogen receptor | 103/107 (96%) | 47/70 (67.0%) | $P<0.001$ |
| Progesterone receptor | 63/107 (59%) | 34/70 (48.5%) | NS |
| pS2-cytoplasmic and membrane | 104/107 (80.0%) | 49/70 (70.0%) | $P<0.001$ |
| pS2-cytoplasmic only | 85/107 (80.0%) | 40/70 (57.0%) | $P<0.001$ |
| Cathepsin D | 96/107 (90.0%) | 34/70 (48.5%) | $P<0.001$ |
| p53 | 9/107 (8.4%) | 26/70 (37.0%) | $P<0.001$ |
| c-*erb*B-2 | 4/107 (3.7%) | 12/70 (17.0%) | $P<0.05$ |

There was a significant difference between these and the mean H-score for the poorly differentiated of 118 ($P$, 0.05, one-way analysis of variance). All of the four mammographically detected carcinomas that were ER negative were infiltrating ductal, one being well differentiated, one moderately and two poorly differentiated. There was no relationship between ER status, H-score and tumour size.

Fifty nine per cent of the mammographically detected carcinomas were positive for PgR, while 48.5% of the control group were positive. The difference between the two groups was not significant. Sixty per cent of the ER-positive mammographically detected carcinomas were PgR positive, and only one tumour was ER negative, PgR positive. The tubular and well-differentiated infiltrating ductal carcinomas were more likely to be PgR positive (79% and 72% respectively). than the moderate (49%) or poorly differentiated (2/6) infiltrating ductal carcinomas ($\chi^2$ 12.68, $P<0.001$).

### *pS2*

Staining was either within the cytoplasm of cells or at the cell membrane, which was predominantly luminal. Carcinomas were considered positive if more than 10% of cells stained. Membrane staining only was observed in 19 mammographically detected carcinomas, cytoplasmic staining only in 44 and a combined pattern in 41. Only three carcinomas were classified as negative if both cytoplasmic and membrane staining was considered, giving a positive rate of 97.0%. If only cytoplasmic reactivity was taken into account, 80% were positive. There was a significant difference from the control group (Table III), which was greater if both staining patterns were considered ($\chi^2$ 26.65, $P<0.001$).

Staining relating only to membranes was seen predominantly in tubular and well-differentiated infiltrating ductal carcinomas. Eleven of the 29 tubular carcinomas had this pattern of staining, and a further 17 tubular carcinomas had both membrane and cytoplasmic reactivity, with only one exhibiting cytoplasmic staining only.

Most tumours were both ER and pS2 positive. Of the four ER-negative tumours all were pS2 positive, and of the three pS2-negative tumours two were ER positive.

### *Cathepsin D*

Only staining of carcinoma cells was considered. The staining indices were grouped as 2–3, weak; 4–5, moderate; 6–7 strong. Reactivity was seen in 96 mammographically detected carcinomas (90%), with 51 (47.6%) having strong staining, 26 (24%) moderate and 19 (18%) weak reactivity. There was a significant difference in the frequency of cathepsin D in the mammographically detected group compared with the control (Table III) ($\chi^2=39$, $P<0.001$). Also, the mammographically detected carcinomas were more likely to stain strongly ($\chi^2$ 41.41, $P<0.001$).

Cathepsin D staining was associated with the size of the tumour, with positive tumours more likely to be <15 mm ($\chi^2$ 7.1, $P<0.01$). There was decreasing positivity with increasing grade, but this was not significant. There was no correlation between ER and cathepsin D.

### *p53*

Nine of the mammographically detected carcinomas had evidence of p53 staining (8.4%) compared with 38.8% in the control group, which was significantly different ($\chi^2$ 22.78, $P<0.001$). All of the positive tumours were moderately (6) or poorly (3) differentiated infiltrating ductal carcinomas. There was no difference in relation to size, using 15 mm as the cut-off point, and only one p53-positive tumour was ER negative.

### *c-erbB-2*

Four of the mammographically detected carcinomas (3.7%) were positive, compared with 12 (17.0%) of the clinical control group (Fisher exact two-tail test, $P<0.05$). All were moderately differentiated infiltrating ductal carcinomas with sizes ranging from 8 to 17 mm, and were ER positive.

### *Proliferation*

The MIB1 index ranged from 1.2 to 59.9 in the mammographically detected group. The overall mean index (11.6) was significantly lower than that for the control group ($t=2.583$, $P<0.05$). The range for this group was 1.0–50.0 with a mean of 15.25.

The mean values for the mammographically detected group increased with histological grade. The mean value for the tubular carcinomas was 7.48 (range 1.8–15.4), for well-differentiated infiltrating ductal carcinomas 9.11 (range 3.5–20.7), for moderately differentiated infiltrating ductal carcinomas 14.75 (range 1.2–59.9), and for poorly differentiated infiltrating ductal carcinomas 26.4 (range 6.7–45.1). The increase was significant (one-way analysis of variance, $P<0.001$). There were no differences in the MIB1 index with increasing size, the key factor within any size range being tumour grade.

There was no relationship between ER H-score and MIB1 index for the mammographically detected group, using the mean score of 11.6 as the cut-off point. Thirty eight per cent (13/34) of the carcinomas with H-scores greater than 200 had a MIB1 index greater than the mean and 31% (22/70) of carcinomas with H-scores between 50 and 200 had a MIB1 index greater than the mean (Table IV). The majority of carcinomas with high score and higher proliferation rates were moderately differentiated.

### Discussion

The aim of mammographic screening is to detect breast carcinomas at an earlier stage of their clinical course when they are of a smaller size and are less likely to have metastasised. Several studies have shown that carcinomas detected by screening programmes have pathological and biological characteristics which are suggestive of a lower malignant potential (Anderson *et al.*, 1986, 1991; Cowan *et al.*, 1991; Klemi *et al.*, 1992). The present study has considered a large number of factors and has confirmed that carcinomas which are detected by mammography differ biologically from carcinomas which present clinically.

The carcinomas studied formed a consecutive series of invasive impalpable tumours. Eighty-nine per cent were 15 mm or less in size, and only 6.5% showed evidence of metastasis. This latter finding is similar to the finding of Ellis *et al.* (1993) for impalpable carcinomas, but lower than that of Anderson *et al.* (1986), who reported metastasis in 22% of cases. The finding of a high proportion of tubular carcinomas (27%) and well-differentiated carcinomas (36% of infiltrating ductal carcinomas) has been reported by others. Anderson *et al.* (1986) found 12% tubular carcinomas and 22.5% variant tubular carcinomas. Cowan *et al.* (1991) classified 46% of all invasive carcinomas as well

**Table IV** Comparison of ER H-score and proliferation index (MIB1, cut-off point = mean value) in relation to type and grade of mammographically detected carcinoma

| | *H-score 50–199* | | *H-score 200 and greater* | |
|---|---|---|---|---|
| | ⩽11.6% | >11.6% | ⩽11.6% | >11.6% |
| Infiltrating ductal | | | | |
| Grade I | 14 | 3 | 6 | 1 |
| Grade II | 12 | 12 | 3 | 10 |
| Grade III | 1 | 2 | 0 | 1 |
| Tubular | 16 | 3 | 12 | 0 |
| Infiltrating lobular | 5 | 2 | 0 | 1 |

differentiated, and Klemi *et al.* (1992) 38%. Ellis *et al.* (1993) found 52% of impalpable carcinomas to be tubular or tubular mixed. Their percentage of tubular and well-differentiated tubular mixed and infiltrating ductal (61%) is very similar to that 63% found in this study. All of these findings differ from those of McKinney *et al.* (1992), who reported only 2.6% (2/77) tubular carcinomas and 14% well-differentiated tumours in a series of impalpable invasive carcinomas.

The rate of ER positivity in this group of impalpable carcinomas is very high in comparison with the control group. The ability to detect ER in fixed, embedded tissue has extended the range of carcinomas that can be studied. Stal *et al.* (1992) studied screen-detected carcinomas but employed a biochemical assay which requires fresh tissue and would not be applicable to impalpable lesions excised under stereotactic control. Their rate was 78%. Soomro *et al.* (1992) used immunohistochemistry to assess screen-detected carcinomas for ER and found 78% to be positive, but their series included palpable and impalpable carcinomas. Of interest in the present study was the finding of a group of carcinomas with high H-scores for ER and high MIB1 (proliferation) indices.

The oestrogen-regulated protein pS2 has a good correlation with ER, both in immunoassays (Foekens *et al.*, 1990) and in immunohistochemical studies (Henry *et al.*, 1991; Thor *et al.*, 1992). It is therefore not surprising that there is a high level of detection in the mammographic group of carcinomas. What is more surprising is the greater level of detection of cathepsin D in the mammographically detected group compared with the clinical controls. This was also found by Cowan *et al.* (1991), but not to the same degree of significance. The higher incidence of cathepsin D staining in these early breast carcinomas seems paradoxical in the light of previous studies which have related cathepsin D to metastasis and shortened disease-free interval (Spyratos *et al.*, 1989; Tandon *et al.*, 1990; Pujol *et al.*, 1993). However, Henry *et al.* (1990) found cathepsin D, as determined immunohistochemistry, to be associated with a better prognosis. The critical factor may well be the cathepsin D in macrophages, which will be measured in immunoassays of tumour homogenates, but can be assessed separately using immunochemistry (Walker *et al.*, 1994).

The finding of a lower incidence of p53 and c-*erb*B-2 in the mammographically detected group compared than in the control group is not surprising since they are associated with poorer differentiation and lack of oestrogen receptor (Walker *et al.*, 1989, 1991; Allred *et al.*, 1992; Poller *et al.*, 1992; Barnes *et al.*, 1993).

The Ki-67 antigen is expressed by cells in the cell cycle (Gerdes *et al.*, 1984). Immunodetection can provide a useful guide to the proliferative status of a carcinoma. The overall mean index of the mammographically detected carcinomas was lower than that of the control group, which is similar to the findings of Klemi *et al.* (1992) for S-phase fraction. Although there was a relationship between MIB1 (Ki-67) index and differentiation, a range of values was found for each of the differentiation categories, with no relationship to size.

The carcinomas detected by mammography are clearly different from the clinically presenting group in many ways. The question arises as to what happens to this impalpable group with time. Linnell *et al.* (1980) suggested that tubular carcinomas may progress to less differentiated carcinomas if left untreated. The tubular mixed carcinomas described by Ellis *et al.* (1992) may lend some support to this contention. Taber *et al.* (1992) proposed that dedifferentiation occurs with increasing size, but the evidence for this is circumstantial. In the present study there were carcinomas 10 mm and less which were moderately and poorly differentiated with no evidence of tubular structures, and no features to suggest a possible origin from a tubular carcinoma. It is therefore more likely that breast carcinomas have several different lines of development and progression, with a proportion of impalpable carcinomas undergoing dedifferentiation with time.

**Acknowledgements**

R Rajakariar undertook these studies while an Intercalated BSc student, with support from the Jean Shanks Foundation. We are grateful to Mrs S Dearing for technical support and Mrs Diana Peters for typing the manuscript.

### References

ALLRED DC, CLARK GM, MOLINA R, TANDON AK, SCHNITT SJ, GILCHRIST KW, OSBORNE CK, TORMEY DC AND MCGUIRE WL. (1992). Overexpression of HER-2/*neu* and its relationship with other prognostic factors change during the progression of in-situ to invasive breast cancer. *Hum. Patho.*, **23**, 974–979.

ANDERSON TJ, LAMB J, ALEXANDER FE, LUTZ W, CHETTY U, FORREST APM, KIRKPATRICK A, MUIR B, ROBERTS MM AND HUGGINS A. (1986). Comparative pathology of prevalent and incident cancers detected by breast screening. *Lancet*, **i**, 519–523.

ANDERSON TJ, LAMB J, DONNAN P, ALEXANDER FE, HUGGINS A, MUIR BB, KIRKPATRICK AE, CHETTY U, HEPBURN W, SMITH A, PRESCOTT RJ AND FORREST P. (1991). Comparative pathology of breast cancer in a radnomised trial of screening. *Br. J. Cancer*, **64**, 108–113.

BARNES DM, DUBLIN EA, FISHER CJ, LEVISON DA AND MILLIS RR. (1993). Immunohistochemical detection of p53 protein in mammary carcinoma: an important new independent indication of prognosis? *Hum. Pathol.*, **24**, 469–476.

BRUUN RASMUSSEN B, ROSE C, THORPE SM, HOU-JENSEN K, DAEHNFELDT JL AND PALSHOF T. (1981). Histopathological characteristics and oestrogen receptor content in primary breast carcinoma. *Virchows Arch. Pathol. Anat.*, **390**, 347–351.

CATTORETTI G, BECKER MHG, KEY G, DUCHROW M, SCHLUTER C, GALLE J AND GERDES J. (1992). Monoclonal antibodies against recombinant parts of the Ki-67 antigen [MIB-1 and MIB-3] detect proliferating cells in microwave processed formalin-fixed paraffin sections. *J. Pathol.*, **168**, 357–363.

CHAMBERLAIN J, COLEMAN D, ELLMAN R AND MOSS SM. (1988). First results on mortality reduction in the UK trial of early detection of breast cancer. *Lancet*, **ii**, 411–416.

COWAN WK, ANGUS B, HENRY J, CORBETT IP, REID WA AND HORNE CHW. (1991). Immunohistochemical and other features of breast carcinomas presenting clinically with those detected by cancer screening. *Br. J. Cancer*, **64**, 780–784.

ELLIS IO, GALEA M, BROUGHTON N, LOCKER A, BLAMEY RW AND ELSTON CW. (1992). Pathological prognostic factors in breast cancer. II. Histological type. Relationship with survival in a large study with long-term follow-up. *Histopathology*, **20**, 479–489.

ELLIS IO, GALEA M, LOCKER A, ROEBUCK EJ, ELSTON CW, BLAMEY RW AND WILSON ARM. (1993). Early experience in breast cancer screening: emphasis on development of protocols for triple assessment. *Breast*, **2**, 148–153.

ELSTON CW AND ELLIS IO. (1991). Pathological prognostic factors in breast cancer. I. The value of histological grade in breast cancer: experience from a large study with long term follow up. *Histopathology*, **19**, 403–40?.

FOEKENS JA, RIO MC, SEGUIN P, VAN PUTTEN WLJ, FAUQUE J, NAP M, KLIJN JGM & CHAMBON P. (1990). Prediction of relapse and survival in breast cancer patients by pS2 protein status. *Cancer Res.*, **50**, 3832–3837.

GERDES J, LEMKE H, BAISCH H, WACHER H-H, SCHWAB V AND STEIN H. (1984). Cell cycle analysis of a cell proliferation-associated human nuclear antigen defined by the monoclonal antibody Ki-67. *J. Immunol.*, **133**, 1710–1715.

HENRY JA, MCCARTHY AL, ANGUS B, WESTLEY BR, MAY FEB, NICHOLSON S, CAIRNS J, HARRIS AL AND HORNE CHW. (1990). Prognostic significance of the oestrogen regulated protein, cathepsin D, in breast cancer. *Cancer*, **65**, 265–277.

HENRY JA, PIGGOTT NH, MALLICK UC, NICHOLSON S, FARNDON JR, WESTLEY BR AND MAY FEB. (1991). pNR-2/pS2 immunohistochemical staining in breast cancer: correlation with prognostic factors and endocrine response. *Br. J. Cancer*, **62**, 615–622.

KLEMI PJ, JOENSUU H, TOIKKANEN S, TUOMINEN J, RASANEN O, TYRKKO J AND PARVINEN I. (1992). Aggressiveness of breast cancer found with and without screening. *Br. Med. J.*, **304**, 467–469.

LINELL F. LJUNBERG O AND ANDERSSON I. (1980). Breast carcinoma aspects of early stages, progression and related problems. *Acta Pathol. Microbiol. Scand.*, Suppl. 272, 63-101.

McCLELLAND RA. WILSON D. LEAKE R. FINLAY P AND NICHOLSON RI. (1991). A multicentre study into the reliability of steroid receptor immunocytochemical assay quantification. *Eur. J. Cancer*, **27**, 711-715.

McKINNEY CD. FRIERSON HF. FECHNER RE. WILHELM MC AND EDGE SB. (1992). Pathologic findings in nonpalpable invasive breast cancer. *Am J. Surg. Pathol.*, **16**, 33-36.

POLLER DN. HUTCHINGS CE. GALEA M. BELL JA. NICHOLSON RA. ELSTON CW. BLAMEY RW AND ELLIS IO. (1992). p53 protein expression in human breast carcinomas: relationship to expression of epidermal growth factor receptor c-*erb*B-2 protein overexpression and oestrogen receptor. *Br. J. Cancer*, **66**, 583-588.

PUJOL P. MAUDELONDE T. DAURES J-P. ROUANET P. BROUILLET J-P. PUJOL H AND ROCHEFORT H. (1993). A prospective study of the prognostic value of cathepsin D levels in breast cancer cytosol. *Cancer*, **71**, 2006-2012.

ROBERTS MM. ALEXANDER FE. ANDERSON TJ. CHETTY V. DONNAN PT AND FORREST APM. (1990). Edinburgh trial of screening for breast cancer: mortality at seven years. *Lancet*, **335**, 241-246.

ROYAL COLLEGE OF PATHOLOGISTS WORKING GROUP. (1990). *NHS Breast Screening Programme: Pathology Reporting in Breast Cancer Screening*. London: Royal College of Pathologists.

SHAPIRO S. VENET W. STRAX P. VENET L AND ROESER R. (1985). Selection, follow-up and analysis in the health insurance plan study: a randomized trial with breast cancer screening. *Natl Cancer Inst. Monogr.*, **67**, 65-74.

SOOMRO S. SHOOSHA S AND SINNET HD. (1992). Oestrogen and progesterone receptors in screen-detected breast carcinoma: an immunohistological study using paraffin sections. *Histopathology*, **21**, 543-547.

SPYRATOS F. MAUDELONDE T. BROUILLET J. BRUNET M. DEFRENNE A. ADRIEN C. HACENE K. DESPLACES A. ROUESSE J AND ROCHEFORT H. (1989). Cathepsin D: an independent prognostic factor for metastasis of breast cancer. *Lancet*, **ii**, 1115-1118.

STAL O. BRISFORS A. CARSTENSEN J. FERRAUD L. HATSCHEK T. NORDENSKJOLD AND THE SOUTH-EAST SWEDEN BREAST CANCER GROUP. (1992). Relationships of DNA ploidy, S-phase fraction and hormone receptor status to tumour stage in breast cancer detected by population screening. *Int. J. Cancer*, **51**, 28-33.

TABAR L. FAGERBERG CJG. GAD A. BALDETORP L. HOLMBERG LH AND GRONTOFT O. (1985). Reduction in mortality from breast cancer after mass screening with mammography. *Lancet*, **i**, 829-832.

TABAR L. FAGERBERG G. DAY NE. DUFFY SW AND KITCHIN RM. (1992). Breast cancer treatment and natural history: new insights from results of screening. *Lancet*, **339**, 412-415.

TANDON AK. CLARK GM. CHAMNESS GC. CHIRGWIN JM AND McGUIRE WL. (1990). Cathepsin D and prognosis in breast cancer. *N. Engl. J. Med.*, **322**, 297-302.

THOR AD. KOERNER FC. EDGERTON SM. WOOD WC. STRACHER MA AND SCHWARTZ LH. (1992). pS2 expression in primary breast carcinomas: relationship to clinical and histological features and survival. *Breast Cancer Res. Treat.*, **21**, 111-119.

VERBEEK ALM. HOLLAND R. STURMANS F. HENDRICKS JHCL. MRAVUNAC M AND DAY NE. (1984). Reduction of breast cancer mortality through mass screening with modern mammography. First results of the Nijmegen project, 1975-1981. *Lancet*, **ii**, 1222-1224.

WALKER RA. GULLICK WJ AND VARLEY J. (1989). An evaluation of immunoreactivity for c-*erb*B-2 protein as a marker of short-term prognosis in breast cancer. *Br. J. Cancer*, **60**, 426-429.

WALKER RA. DEARING SJ. LANE DP AND VARLEY JM. (1991). Expression of p53 protein in infiltrating and in-situ breast carcinoma. *J. Pathol.*, **165**, 203-211.

WALKER RA. DENLEY H AND DOOKERAN KA. (1994). Cathepsin D in breast carcinomas-the role of the stromal cell component. *Oncology Rep.*, **1**, 227-231.